# Development of fragment-specific osteopontin antibodies and ELISA for quantification in human metastatic breast cancer

**DOI:** 10.1186/1471-2407-8-38

**Published:** 2008-01-31

**Authors:** Alicia Plumer, Hongyi Duan, Sripriya Subramaniam, F Lee Lucas, Susan Miesfeldt, Ah-Kau Ng, Lucy Liaw

**Affiliations:** 1 Dept. Applied Medical Sciences, Univ. Southern Maine, P.O. Box 9300 Portland, ME 04104, USA; 2 Center for Molecular Medicine, Maine Medical Center Research Institute, 81 Research Drive, Scarborough, ME 04074, USA; 3 Center for Outcomes Research and Evaluation, Maine Medical Center, Portland, ME 04101, USA; 4 Maine Center for Cancer Medicine and Blood Disorders, 100 Campus Drive, Scarborough, ME 04074, USA

## Abstract

**Background:**

Osteopontin (OPN) is associated with human cancers, and circulating blood OPN may have diagnostic or prognostic value in clinical oncology.

**Methods:**

To evaluate OPN as a cancer biomarker, we generated and characterized five novel mouse monoclonal antibodies against the human full-length OPN (fl-OPN). Epitopes recognized by four antibodies (2C5, 2F10, 2H9, and 2E11) map to N-terminal OPN (aa1-166); one (1F11) maps to C-terminal OPN (aa167-314). These antibodies recognize recombinant and native OPN by ELISA and immunoblot, cross reacting with human and mouse OPN. Two of these novel antibodies (2F10 and 1F11) were used to develop a quantitative enzyme linked immunosorbent assay (ELISA) for fl-OPN.

**Results:**

In comparison with commercially available ELISAs, our assay had high accuracy in measuring fl-OPN standards, and high sensitivity. Specifically, our ELISA has a linear dose response between 0.078 ng/ml-10 ng/ml, with a sensitivity of 13.9 pg/ml. We utilized this assay to quantify fl-OPN in the plasma of healthy volunteers in comparison with patients with metastatic breast cancer. The average circulating plasma fl-OPN in healthy volunteers was 1.2 ng/ml, compared to 4.76 ng/ml in patients with metastatic breast cancer (p = 0.0042). Although the increase in fl-OPN in cancer patients is consistent with previous studies, the measured quantity varied greatly between all existing fl-OPN ELISAs.

**Conclusion:**

Because OPN is a complex molecule with diversity from alternative splicing, post-translational modification, extracellular proteolytic modification, and participation in protein complexes, we suggest that further understanding of specific isoform recognition of multiple OPN species is essential for future studies of OPN biomarker utility.

## Background

Osteopontin (OPN) is a secreted phosphorylated glycoprotein that was originally isolated from bone and controls biomineralization, osteoclast differentiation, and bone resorption [1]. Recent literature has linked up-regulated expression of osteopontin with cancer, atherosclerosis, bone remodeling, angiogenesis, wound healing and tissue injuries, as well as certain pathologies such as restenosis, formation of kidney stones, and autoimmune disease [2-7]. OPN belongs to the SIBLING (Small integrin binding ligand N-linked glycoprotein) protein family, and binds receptors including integrins and CD44.

OPN is of high interest in human cancer, and is expressed in malignancies of various tissue origins [8,9]. Since OPN is a secreted molecule that is found in the circulation and in bodily fluids, it has been explored as a potential non-invasive biomarker for the diagnosis or progression of cancer. Early studies of OPN identified it as a highly phosphorylated protein associated with advanced-stage cancers [10,11] that was found as lower molecular weight fragments in human serum, suggesting proteolysis by enzymes related to the coagulation cascade. Several studies have used enzyme-linked immunosorbent assays (ELISA) as a method to quantify circulating blood OPN levels in cancer patients. While the studies yielded variable conclusions, levels of circulating plasma OPN may be a biomarker of ovarian cancer [12], as there was a trend for decreased OPN levels following treatment of ovarian cancer, and levels rose early in patients with recurrent disease [13]. High levels of OPN were also present in the blood of patients with lung carcinoma [14], hepatocarcinoma [15], metastatic prostate carcinoma [16], and metastatic breast cancer [17], compared to healthy volunteers. Levels of circulating OPN have also been studied as a prognostic factor, and high OPN levels are associated with poor prognosis in esophageal carcinoma [18], head and neck squamous cell carcinoma [19], and breast cancer [20].

ELISA quantification of blood OPN has been accomplished using commercially available assays from IBL and Assay Designs. One recent study directly compared these assays to quantify plasma OPN from patients with head and neck cancer or cervical cancer. The quantification of the same samples were positively correlated, although the absolute values were significantly different, suggesting that assay accuracy is a challenge [21]. There is also an ELISA test from R&D Systems (Quantikine) available commercially. We have developed and characterized a novel ELISA detecting full-length OPN from human plasma, and find a greater sensitivity to low OPN concentrations in our assay compared to the existing assays. However, our ELISA also varies in absolute values with the IBL and Assay Designs OPN ELISAs, although our results were more accurate, along with the R&D ELISA, in measurement of commercially available OPN protein standards. Our quantification of OPN levels in plasma from healthy individuals versus those with metastatic breast cancer patients showed an elevated level among those with breast cancer. Of interest, our results also demonstrated significantly different absolute values (ng/ml) in both populations compared to previously published literature. We conclude that existing assays for measurement of OPN in human blood have not been independently validated, and that there may be complications in quantification of OPN from complex samples, possibly due to interacting proteins found in human plasma that may affect accurate OPN quantification.

## Methods

### OPN protein and monoclonal antibody production

Recombinant human full-length OPN (fl-OPN), human N-OPN and human C-OPN fragments representing the MMP cleaved products were produced in *E. coli*, and purified as previously described [22]. OPN trilevel controls were purchased from R&D systems. This recombinant full-length OPN has a C-terminal 6x-His tag and was expressed in NS0 mouse myeloma cells. The ranges given for these controls are 1.66–2.26 ng/ml, 5.03–6.57 ng/ml, 10.13–13.09 ng/ml. Native OPN purified from human milk [23] was a generous gift of Esben Sørensen (University of Aarhus, Denmark). All animal protocols were reviewed and approved by our Institutional Animal Use and Care Committee, and performed under veterinary supervision. The fl-OPN protein was used for immunization of OPN homozygous null mice [24]. Prior to immunization, pre-immune blood was collected from mice by retro-orbital bleed. Antibody generation was accomplished by subcutaneous injection of 100 μg fl-OPN in Complete Freund's adjuvent (Sigma, F5881), followed by subsequent injections of 50 μg fl-OPN in Incomplete Freund's Adjuvent (Sigma F5506) on days 13, 33, 45, and 161 days after the initial injection. The mice were then bled and antibody titer measured by ELISA. Final injections of 30 μg fl-OPN intraperitoneally/20 μg fl-OPN subcutaneously were given 4 days before collection of the spleen for fusion. The hybridomas were generated by fusing splenocytes with FO myeloma cells of Balb/c origin (ATCC, Manassas, VA), according to a standard protocol developed by Kohler and Milstein (Kohler G, Milstein, C, 1975), with minor modifications.

### Enzyme-linked immunosorbent assay (ELISA)

After five rounds of immunization, 0.1 ml blood was collected by retro-orbital bleed to determine the OPN antibody level in the serum. A 96-well microtiter plate (BD Biosciences, Bedford, MA.) was coated with 50 ng/ml (100 μl/well) of purified human fl-OPN and incubated at 4°C overnight. The plate was blocked with 3% nonfat dry milk in PBS-T (PBS pH = 7.3, 0.05% Tween-20) at room temperature for 2 hours. The sample serum was loaded at a 1:100 dilution in 3% nonfat dry milk/PBS-T, followed by serial 5-fold dilutions. After incubation at 37°C for 1 hour, the plate was washed with PBS-T, and incubated at 37°C for 2 hours with a combination of two separate secondary antibodies at 100 μl/well: 1:5000 dilution of HRP-conjugated goat anti-mouse IgG, Fcγ fragment specific, and a 1:5000 dilution of HRP-conjugated AffiniPure goat anti-mouse IgG + IgM (H+L) (both from Jackson ImmunoResearch Laboratories). Antigen was detected by incubation with 100 μl/well of tetramethylbenzidine for 15 minutes, and the reaction was stopped by 100 μl/well of 1 M HCl. The plate was read at 405 nm using an automated microplate reader (Bio-Tek Instruments). Screening of subsequent hybridomas was performed similarly, with HRP-conjugated goat antimouse IgG + IgM used as the secondary antibody. In addition, hybridoma screening included negative selection of clones that recognized an unrelated control his-tagged protein, and positive selection of clones recognizing a second recombinant OPN without a his epitope tag. For crossreactivity studies, wells were coated with either human or mouse fl-OPN, and human N- and Cterminal OPN fragments were used for epitope mapping. To compare epitope specificity, unlabeled anti-OPN antibodies were used to compete with biotinylated 1F11 and 2F10 for binding to specific OPN epitopes. Unlabeled antibodies were incubated first, followed by reaction with the biotinylated antibodies to detect binding.

### Production of ascites fluid and antibody purification

Ascites fluid was collected using either Hsd athymic nude mice or F1 hybrids (H-2^b/d ^MHC class) of OPN heterozygous mice (C57BL/6 H-2^b^) crossed to Balb/c mice (H-2^d^). Mice were primed with an intraperitoneal injection of incomplete Freund's adjuvant, followed by injection of 5 × 10^5 ^– 5 × 10^6 ^hybridoma cells. Ascites was collected from peritoneal tapping. For antibody purification, ascites were centrifuged at 14,000 *g *for 15 minutes and diluted with 5 volumes of 20 mM sodium phosphate buffer, pH = 7.0. Antibodies were purified over a protein G column (Sigma), and eluted with 0.1 M glycine-HCl, pH = 2.7 and adjusted to neutral pH with 1 M Tris-HCl, pH = 8. Eluted antibodies were dialyzed against PBS. Antibody concentration was determined with a DC protein assay kit (BioRad). On average, 1–4 mg antibody was purified from 1 ml ascites fluid. Purity of antibodies was assessed by SDS-PAGE and Coomassie blue staining. Antibody isotypes were determined using the IsoStrip assay (Roche) following the manufacturer's instructions.

### Antibody biotinylation

Sulfo-NHS-LC-Biotin [sulfosuccinimidyl-6-(biotinamido) hexanoate] (MW 556.59, Pierce) was prepared at 10 mg/ml in PBS and added to dialyzed MAbs 1F11, 2F10, and 1E3 at a ratio of 186 μg, 148 μg and 150 μg of biotin per mg of antibody, respectively. IE3, a monoclonal antibody against platelet activation antigen CD62-P was used as a control [25]. The mixtures were incubated at room temperature for 1 hour before being dialyzed extensively against PBS to remove uncoupled biotin. The quantities of the biotinylated proteins were then determined using a DC protein assay kit (Bio-Rad).

### Sandwich ELISA

A two-antibody sandwich ELISA was developed as a quantitative ELISA for fl-OPN measurement. MAb 2F10, which binds specifically to the N-terminus of OPN, was selected as the capture antibody; while 1F11, specific for the C-terminus, was selected as the detection antibody (1F11 was biotinylated). This assay design aimed to detect only OPN molecules that were intact and not cleaved. Firstly, chessboard titrations were performed with 2F10 as the capture antibody and 1F11b as the detecting antibody and fl-OPN as the antigen. The chessboard showed that a dilution of 1.5625 μg/ml of 2F10 and 0.0975 μg/ml of 1F11b would be the optimal concentrations for the sandwich ELISA.

The capture antibody, 2F10, was then used at a concentration of 1.5625 μg/ml in PBS pH = 8.0 and coated at 100 μl/well overnight at 4°C. PMSF (1 mM) and protease inhibitor cocktail (Sigma, 0.5 μl/ml) were added to the blocking and carrying solution of 1% BSA/PBS (referred to hereafter as 1%BSA/PBS/PIC). Wells were blocked with 1%BSA/PBS/PIC for 2 hours at room temperature on a plate shaker at ~500 rpm, washed, and samples and standards loaded for 1 hour at room temperature. After washing, the biotinylated detecting antibody, 1F11b, was added at 0.0975 μg/ml in 1%BSA/PBS/PIC and incubated for 1 hour. After washing, 1:2000 HRPconjugated streptavidin (Jackson Immunoresearch) was incubated for 1 hour, followed by washing and antigen detection with tetramethylbenzidine (TMB). The chromogenic reaction was stopped with 1 M HCl and the plate read at 405 nm with a correction at 570 nm.

### Plasma collection

Our Institutional Review Board evaluated and approved the protocols for the use of healthy volunteer and patient samples. Plasma samples were collected from 55 healthy blood donors as well as 40 women with metastatic breast cancer (Dr. Susan Miesfeldt, Maine Center for Cancer Medicine and Blood Disorders, Scarborough, Maine). All samples were collected in EDTA tubes; the plasma was then separated, aliquoted into 200 μl and frozen at -70°C within 4 hours of collection. Data on initial stage, histology, receptor status, and disease free interval are presented in the text.

### Immunoblot analysis

Sodium dodecyl sulfate-polyacrylamide gel electrophoresis (SDS-PAGE) was performed with a Mini-PROTEAN Gel Electrophoresis Unit (Bio-Rad). Protein samples were diluted appropriately and boiled for 5 minutes in the sample buffer (63 mM Tris, 2% SDS, 0.01% bromophenol blue, 5% β-mercaptoethanol). The resolving gel was 12.5% and the stacking gel was 5%. The running buffer consisted of 25 mM Tris base, 192 mM glycine, 0.1% SDS, pH = 8.8. The separated proteins on the gel were transferred to a polyvinylidene diflouride (PVDF) membrane with the Mini-PROTEAN Gel Electrophoresis unit (Bio-Rad Laboratories) The transfer buffer consisted of 25 mM Tris, 192 mM glycine and 0.1% SDS, pH = 8.3.

Nonspecific binding was blocked with 3% BSA in TBS-T buffer (10 mM Tris, pH = 8.0, 150 mM NaCl, 0.05%Tween-20) at room temperature for 1 hour. The membrane was then cut into strips and incubated in individual tubes with the appropriate dilution of the MAbs in 3% BSA in TBST buffer overnight. After the overnight incubation at 4°C the strips were washed in TBS-T in their own tubes for 1.5 hours. The strips were incubated with 3% BSA for 30 minutes, washed 3X in TBS-T, and incubated with HRP-conjugated goat anti-mouse IgG, Fcγ (1:10,000) in 3% BSA TBS-T for 1 hour, followed by a TBS-T and 3% BSA washes. The bands were visualized using an ECL Western blotting detection reagent (Amersham). Prestained molecular weight standards (Bio-Rad) were used to estimate protein sizes.

## Results

### Characterization of anti-OPN monoclonal antibodies

**Figure 1:**
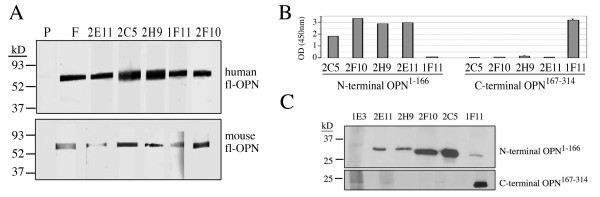
**Characterization of anti-OPN MAb**. All Mab were screened based on their ELISA detection of full-length human OPN (fl-OPN). A) Recombinant human or mouse fl-OPN were used for immunoblot with the anti-OPN MAb indicated. Each lane was loaded with 20 ng OPN protein for detection. While the recognition of human OPN was greater by all MAb, they all cross-reacted with murine OPN. B) An ELISA was performed using the human N-terminal (aa-1-167) or C-terminal (aa167-314) human recombinant fragments. Each lane was loaded with 250 ng protein. MAb 1E3 is an irrelevant antibody used as a negative control. 2C5, 2H9, 2F10, and 2E11 recognize OPN epitopes on the N-terminal fragment, while 1F11 recognizes an epitope on the C-terminal fragment. C) The same OPN fragments were tested by immunoblot, and yielded consistent results with the ELISA.

Monoclonal antibodies (MAb) recognizing human fl-OPN were purified and characterized, and included MAb 2C5, 2H9, 2F10, 2E11 and 1F11. Antibodies were originally identified by their recognition of fl-OPN by ELISA. All are of the IgG1 subtype, with a κ light chain. During the course of the screening, these antibodies were found not to cross react with a random hiscontaining protein, and also recognize a recombinant OPN lacking a his epitope tag. Using human or mouse recombinant fl-OPN protein, we found that under denaturing, reduced conditions, all MAb recognized the recombinant proteins, although the detection of human OPN was more robust (Fig. 1A). OPN is known to run anomalously by SDS-PAGE (~50–70 kD), not corresponding to its actual molecular mass (~35 kD). Antibody recognition to the OPN molecule was mapped to either an N-terminal or C-terminal epitope by ELISA, using purified recombinant protein (Fig. 1B). Epitopes of MAb 2C5, 2F10, 2H9, and 2E11 map to the human N-terminal fragment (aa1-aa166), while that of 1F11 maps to the C-terminal fragment (aa167-aa314). This was verified by immunoblot analysis using the same recombinant N-terminal or C-terminal OPN fragment (Fig. 1C).

**Figure 2:**
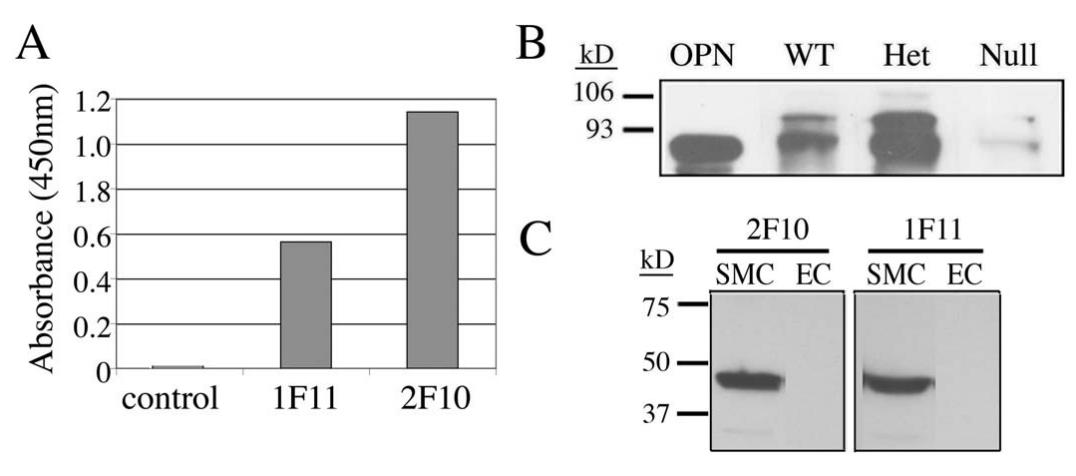
**Recognition of native OPN by anti-OPN MAb**. A) Human milk OPN was used in ELISA, and 1F11 and 2F10 binding compared to an irrelevant antibody (MAb 1E3, control). B) Whole kidney lysates were collected from wild type (WT) mice, OPN heterozygous mice (Het), and OPN null mutant mice (Null). Lysates were loaded equally and analyzed by SDS-PAGE followed by immunoblotting using 1F11. Recombinant OPN was used as a control. C) Human aortic smooth muscle cells (SMC) were transduced with activated Notch1 receptor [41] to increase OPN expression, and compared to human umbilical vein endothelial cells (EC). Both 2F10 and 1F11 recognize OPN in SMC.

The antibodies were then tested for recognition of native OPN protein by ELISA and immunoblot analysis. We obtained purified native human milk OPN [23] and tested activity of the MAb by ELISA (Fig. 2A). MAb 1F11 and 2F10, which we subsequently used to develop the quantitative sandwich ELISA, both recognized human native milk OPN. We also collected mouse kidneys, which are tissues known to contain endogenous OPN protein [26,27]. We tested wild kidneys from wild type, OPN heterozygous, and OPN null mice [24], compared to recombinant human OPN (Fig. 2B). OPN was detected in kidneys from wild type and OPN heterozygous mice, but not from kidneys from OPN null animals, showing recognition of mouse native OPN. Finally, since OPN expression in smooth muscle cells is induced by Notch signaling [28], we also tested human aortic smooth muscle cells with activated Notch, and found strong expression of OPN detectable by both 1F11 and 2F10 MAb. These data support the specificity of the antibody in recognition of native mouse and human OPN protein.

### Development of OPN quantitative enzyme-linked immunosorbent assay (ELISA)

MAb 2F10 specific for the N-terminus of OPN and MAb 1F11 specific for the C- terminus were evaluated as a capture-detection antibody pair in a sandwich ELISA for OPN quantification. To aid detection of signals, the detection antibody was biotinylated. 2F10 was designated the capture antibody, and biotinylated 1F11 was the detection antibody. This antibody selection ensured that only relatively intact OPN molecules would be recognized and quantitatively determined. Chessboard reagent titration experiments were carried out to define optimal assay parameters. Based on this analysis (Table 1) using excess antigen, we found that absorbance plateaued from 50 μg/ml to 3.125 μg/ml of capture antibody, with the drop occurring using 0.78 μg/ml. Thus, we determined the optimal capture antibody concentration as 1.56 μg/ml. In the second checkerboard assay, 1.56 μg/ml was used as the coating concentration with a serial dilution of fl-OPN from 0.156 μg/ml to 2.4 pg/ml, with no fl-OPN as a negative control. From columns 1–11 a serial dilution of detecting antibody was used from 1.56 μg/ml to 1.5 ng/ml, with no detecting antibody in column 12. At 9.7 ng/ml of fl-OPN, the absorbance plateaued between 1.56 μg/ml of 1F11b to 0.195 μg/ml, with a drop occurring at 0.098 μg/ml. A titration of the fl-OPN antigen occurs using 0.098 μg/ml 1F11b between 19.5 ng/ml of Fl-OPN to 2.4 pg/ml. Thus, the capture antibody optimum concentration is 1.56 μg/ml while the detecting antibody 1F11b optimum concentration is 0.0975 μg/ml.

**Table 1:** Chessboard titrations of ELISA antibodies

	2F10 μg/ml											
fl-OPN	50	25	12.5	6.25	3.125	**1.56**	0.781	0.39	0.20	0.098	0.049	0
5	2.66	2.73	2.92	3.32	3.66	**3.78**	1.30	0.76	0.54	0.43	0.459	0.332
2.5	2.84	o.s.	3.07	o.s.	o.s.	**3.89**	1.16	0.60	0.40	0.29	0.276	0.18
1.25	2.62	o.s.	o.s.	3.44	o.s.	**3.95**	1.14	0.49	0.32	0.20	0.174	0.119
0.625	2.74	o.s.	o.s.	o.s.	o.s.	**o.s.**	1.17	0.34	0.24	0.17	0.154	0.086
0.313	2.86	o.s.	o.s.	o.s.	o.s.	**o.s.**	1.01	0.31	0.20	0.15	0.126	0.074
0.156	o.s.	o.s.	o.s.	o.s.	o.s.	**o.s.**	0.964	0.322	0.184	0.106	0.076	0.054
**0.078**	**o.s.**	**o.s.**	**o.s.**	**o.s.**	**o.s.**	**o.s.**	**0.971**	**0.304**	**0.182**	**0.102**	**0.076**	**0.05**
0	1.619	1.869	1.071	0.523	0.285	**0.177**	0.119	0.084	0.073	0.071	0.069	0.071

	1F11b μg/ml											

fl-OPN	1.56	0.78	0.39	0.195	**0.098**	0.049	0.024	0.012	0.006	0.003	0.0015	0
0.156	3.66	3.80	3.801	3.77	**3.74**	3.61	2.16	1.473	0.77	0.41	0.254	0.041
0.078	3.83	o.s.	o.s.	3.93	**o.s.**	3.31	1.91	1.06	0.62	0.37	0.231	0.041
0.039	3.83	o.s.	o.s.	o.s.	**3.96**	3.07	2.36	1.047	0.56	0.32	0.185	0.035
0.020	3.84	o.s.	o.s.	o.s.	**3.76**	2.43	1.41	0.763	0.41	0.24	0.154	0.039
0.0098	3.86	o.s.	3.48	3.49	**2.45**	1.61	0.95	0.643	0.34	0.20	0.132	0.039
0.0049	3.82	3.81	2.79	2.27	**1.53**	0.96	0.50	0.358	0.22	0.16	0.142	0.036
**0.002**	**3.05**	**2.40**	**1.54**	**1.18**	**0.90**	**0.60**	**0.32**	**0.215**	**0.13**	**0.087**	**0.07**	**0.04**
0	0.46	0.26	0.17	0.10	**0.069**	0.05	0.05	0.04	0.03	0.04	0.038	0.038

Both the capture antibody (2F10, top) and the detecting antibody (1F11, bottom) were analyzed for dose response in a checkerboard analysis. The optimal concentrations (bold) are 1.56 μg/ml capture antibody and 0.098 μg/ml detecting antibody 1F11b. Conditions listed as o.s. (off-scale) had an absorbance > 4.0.

**Figure 3:**
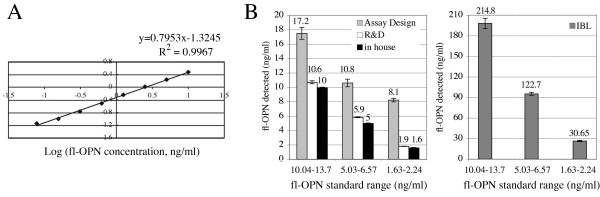
**Comparison of OPN ELISA tests**. A) Our assay was tested for quantification of recombinant fl-OPN using monoclonal antibodies 2F10 and 1F11-biotin. 2F10 reacts to an epitope on the N-terminal of human OPN and 1F11-biotin reacts with an epitope on the C-terminal side of human OPN. The optical density of the color product is proportional to the quantity of human OPN. The measuring range is from 78 pg/ml to 10 ng/ml. B) Commercially prepared human OPN standards of high (10.1 ng/ml-13.1 ng/ml), medium (5 ng/ml-6.6 ng/ml) or low (1.7 ng/ml-2.3 ng/ml) concentrations were used in four ELISA tests: our in-house test, and those commercially available from Assay Designs, R&D Systems (Quantikine) and IBL. The IBL results are graphed separately because the IBL quantification yielded values that were extremely high compared to the other assays and the standard ranges.

A sandwich ELISA was developed using MAb 2F10 (N-terminal OPN specific) as capture antibody and biotinylated 1F11 (C-terminal OPN specific) as detection antibody. This ELISA permits quantitative, rapid and reproducible measurement of OPN levels in blood plasma and other bodily fluids. The fl-OPN was used as antigen in this assay to generate the standard curve for calculating OPN concentration of unknown samples. The resulting standard curve had a linear correlation (R^2^>0.99, Fig. 3A) between OPN concentration and A_405nm_, with a linear doseresponse range of 0.078 ng/ml-10 ng/ml. Thus, the sandwich ELISA generates a standard curve that can be used to provide accurate determinations of fl-OPN concentrations in test samples. The assay protocol was followed subsequently to measure OPN level in test samples. Assay sensitivity was calculated by comparing binding with no fl-OPN compared to 0.156 ng/ml. The detection limit was determined as the concentration of human OPN measured at two standard deviations from 0 pg/ml standard along the curve. The mean OD for the control was 0.049 ± 0.0017, and the mean OD at 0.156 ng/ml was 0.087 ± 0.0025, giving a deltaOD = 0.038. Our calculations yielded a sensitivity of 13.9 pg/ml.

### Comparison of fl-OPN ELISAs

Currently there are three fl-OPN kits on the market (IBL, Assay Designs, R&D Systems) used previously to quantify plasma OPN levels in cancer patients. Thus, we compared our ELISA with the commercially available tests. The detection limits of these assays are: IBL, 5–320 ng/ml; Assay Designs, 2–32 ng/ml; Quantikine R&D, 0.312–20 ng/ml. We obtained fl-OPN control proteins from R&D Systems representing a high (10.1–13.1 ng/ml), middle (5–6.6 ng/ml), and low (1.7–2.3 ng/ml) protein concentration, and compared assay results using these standards (Fig. 3B). The quantification of fl-OPN varied in all assays, and we compared the actual values with the known standards, as well as the relationship between the three standards, which represent a twofold (1:2) and three fold (1:3) decrease from the high and the middle standards, respectively. Our in-house assay and the Quantikine R&D ELISA were the most accurate, while the Assay Designs over-estimated the standard values from 1.2-fold to 3.6 fold. The relationship between the standards from highest to lowest (1:2/1:3) was proportionally consistent with the in-house (1:2/1:3.1) and Quantikine R&D (1:1.8/1:3.1) assays, but not with the Assay Designs kit (1:1.6/1:1.3). The IBL ELISA kit gave extremely high measurements for these same standards, running 13-fold to 18-fold higher than the standard ranges. The relationship between the high and middle standards was measured as expected with the IBL assay (1:1.8), however, was not consistent between the middle and low standard (1:4).

### Measurement of OPN in Plasma Samples

**Figure 4:**
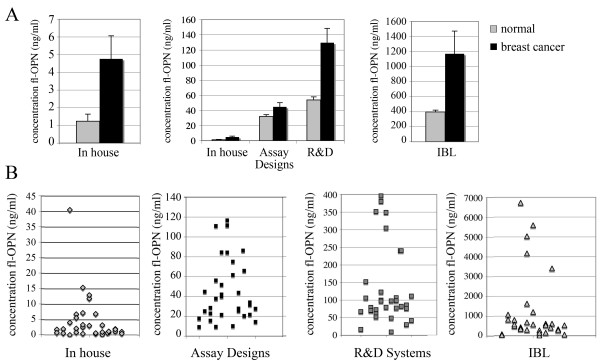
**Quantification of circulating fl-OPN in plasma from breast cancer patients compared to normal healthy volunteers**. A) Our newly developed ELISA (MAbs 2F10 and 1F11) were used to quantify plasma fl-OPN levels in fifty healthy volunteers (normal) versus forty patients with metastatic breast cancer (Stage IV). A significant increase was found in the cancer patient population. Graphed are means ± SEM. The same samples from normal volunteers or patients with metastatic breast cancer were quantified using the different fl-OPN assays, and graphed as means ± SEM. Note the different y-axis scales. The data are shown individually as well to reflect the range of values (B).

In an initial attempt to evaluate OPN as a biomarker for cancer, the quantitative ELISA was used to measure fl-OPN levels in patients with metastatic breast cancer (Table 2). In addition, because the available fl-OPN assays were so variable, we compared our results to those using the commercially available kits. The patient population with metastatic breast cancer was chosen for testing, since similar populations have previously been studied with the commercially available OPN ELISAs, and provide a basis for comparison. Using our in-house assay, we initially compared fl-OPN levels in fifty-one healthy controls with levels from forty metastatic breast cancer patients (Fig. 4A). The plasma fl-OPN level among control subjects averaged 1.22 ± 0.446 ng/ml, while the average level in breast cancer samples was 4.76 ± 1.233 ng/ml (p = 0.0042).

**Table 2:** Cancer patient study population.

**Description**	**n**	**%**
**Histology**		
Invasive ductal	27	68
Invasive lobular	2	5
Other	11	28
**Presenting stage of disease**		
I	5	13
II	15	38
III	8	20
IV	8	20
Unknown	4	10
**Disease free interval**		
0	11	28
1–9 yrs	19	48
10+ yrs	9	23
Unknown	1	3
**Hormone receptor status**		
ER+/PR+	25	63
ER+/PR-	8	20
ER-/PR+	1	3
ER-/PR-	3	8
Unknown	3	8
***HER-2*/*neu *overexpression**		
Positive	4	10
Negative	27	68
Unknown	9	23

Patient characteristics at time of breast cancer diagnosis (n = 40). Abbreviations: PR, progesterone receptor; ER, estrogen receptor

We then compared our results of fl-OPN quantification in the plasma of normal volunteers or breast cancer patients with the commercially available assays. In all cases, the standard curves were linear, with R^2 ^values of 0.99 (Assay Designs), 0.99 (in-house test), 0.98 (R&D), and 0.98 (IBL). As we suspected, the average values obtained for fl-OPN levels were highly variable between assays using the same group of breast cancer patient samples (Fig. 4). Consistent with the prior comparison with the recombinant fl-OPN standards (Fig. 3B), the IBL assay reflected the highest amount of fl-OPN, with an average of 1170 ng/ml, which is 10-fold higher than the results from the other three assays (Assay Designs 44.7 ng/ml, in house 4.3 ng/ml, R&D 129 ng/ml). Surprisingly, however, the in-house assay and the R&D assay, which were concordant in accurately quantifying the recombinant standards (Fig. 3B), were significantly different, with the quantification from the R&D assay yielding values more than 10-fold higher than the in-house ELISA. Also unexpected was the observation that the Assay Design test quantification was intermediate between the in-house and the R&D assay, since it showed consistently higher values than the other two using the recombinant fl-OPN standards. There was heterogeneity in OPN levels in the breast cancer patients (Fig. 4B). Similarly, quantification of plasma OPN in normal healthy volunteers ranged between assays used. In general, the relationship followed the breast cancer plasma quantification, with the in house yielding the lowest mean value, (1.2 ng/ml), followed by Assay Designs (32 ng/ml), R&D (53.9 ng/ml), and IBL (396.3 ng/ml). Using all assays, the amount of circulating plasma OPN was greater in the breast cancer samples compared to those from normal volunteers (Assay Designs p = 0.025, R&D p = 0.0001, IBL p = 0.006). These results show that OPN is difficult to accurately quantify in human blood, and complicating factors need to be considered when evaluating ELISA data quantifying fl-OPN.

## Discussion

Biomarkers have been extensively studied across the broad array of events that lead to the initial development and ultimate progression of breast cancer. The present use of breast cancer-related biomarkers is largely limited to: 1) prognosis among those with a newly diagnosed cancer; 2) assessment of an anticipated response to therapy and 3) monitoring for those who are actively undergoing treatment for known locally advanced or metastatic disease. There is a widely recognized need for biomarkers that could improve the clinician's ability to assess a woman's risk for breast cancer as well as his/her ability to detect breast cancer at it's earliest stages and to further identify therapeutic targets [29,30].

A limited number of studies have tested the utility of fl-OPN as a circulating breast cancer biomarker, using an ELISA assay. In the first study [17], patients with metastatic breast cancer disease were compared to control subjects, and additionally, subdivided into groups with increasing evidence of metastatic spread. The levels of plasma osteopontin in the cohort with metastatic disease was significantly increased compared to controls (p < 0.0001), and additionally, fl-OPN levels were further increased in patients with three or more sites of metastatic involvement compared to one or two metastatic sites. Interestingly, follow up studies demonstrated that increased plasma fl-OPN was associated with shorter survival of patients (p < 0.001).

That compelling study was validated by a prospective study in metastatic breast cancer [20], in which 158 newly diagnosed women with metastatic breast cancer were followed every 3 to 12 weeks, during and after therapy until death. Elevated baseline (newly diagnosed) fl-OPN levels correlated with short survival (p = 0.02), and an increase in fl-OPN levels during the course of the study had the most prognostic value for poor survival (p = 0.0003). This study demonstrated that sequential monitoring of fl-OPN over time may provide data to indicate the course and duration of the disease, and may assist in clinical therapy decisions. Similar conclusions were drawn from a study that examined OPN by immunostaining of tumors from breast cancer patients [31].

There are several issues that are still outstanding with regards to fl-OPN as a biomarker. First, Vordemark et al. [21] highlighted the issues with variation between fl-OPN ELISA test kits, and we also found a similar problem in direct comparison of our newly developed ELISA with commercially available kits. Therefore, an accurate quantification of fl-OPN has not been independently validated, and based on our tests with known standard ranges of fl-OPN, it appears that previous quantification of circulating fl-OPN may have overestimated levels. It is important to note differences between the ELISAs compared in this study (Table 3). The IBL kit uses a polyclonal antibody against a peptide representing aa17–31 as the capture antibody, and a monoclonal antibody recognizing an epitope on the right side of the thrombin cleavage site (between aa168–169); the antigen was recombinant human fl-OPN. The R&D Systems used recombinant fl-OPN protein to generate a mouse monoclonal (undefined) and a rabbit polyclonal (undefined) for the capture and detection antibody, respectively. The Assay Designs test has a capture antibody that is a monoclonal antibody raised against a peptide (aa162–172) spanning a major MMP site (aa166–167) and the thrombin cleavage site (between aa168–169), and monoclonal antibody against the C-terminal fragment as the detecting antibody. Finally, our newly developed assay uses two monoclonal antibodies raised against recombinant fl-OPN, one recognizing the N-terminal fragment as the capture antibody, and one recognizing the C-terminal fragment as the detecting antibody.

**Table 3:** Comparison of fl-OPN ELISA

	**IBL**	**Assay Designs**	**R&D Quantikine**	**In house**
**Capture antibody**	Anti-human OPN (O-17), rabbit polyclonal IgG, recognizes N-terminal ^17^IPVKQADSGSSEEKQ	Mouse monoclonal antibody to OPN, epitope includes ^162^SVVYGLRSKSK	Mouse monoclonal antibody to OPN	Mouse monoclonal anti-human OPN to N-fragment
**Antigen**	Untagged rOPN/CHO cells	6x-C-terminal His tagged rOPN/mouse NS0 cells	6x-C-terminal His tagged rOPN/mouse NS0 cells	6x-C-terminal his tagged rOPN/*E. coli*
**Detecting antibody**	Anti-human OPN (10A16), mouse IgG, Fab'-HRP, recognizes to the right of thrombin cleavag site ^170^KSKKFRRPDIQYPDATDE	Biotinylated monoclonal antibody to OPN. Epitope is located after the thrombin cleavage site,	Polyclonal anti-OPN conjugated to HRP	Mouse monoclonal anti-human OPN to C-fragment
**Detection and system**	Full-length OPN, HRP-TMB	Full-length OPN, biotin-streptavidin-AP	Full-length OPN, HRP-TMB	Full-length OPN, biotin-streptavidin-HRP
**Measur range**	5–320 ng/ml	2–32 ng/ml	0.31–20 ng/ml	0.078–10 ng/ml
**Sensitivy**	5–320 ng/ml	0.110 ng/ml	0.011 ng/ml	0.014 ng/ml

Characteristics of each assay are indicated.

Because the IBL test uses a polyclonal antibody as capture antibody and the R&D Systems test has a polyclonal detecting antibody, it is possible that there is more wide recognition of epitopes including potential post-translational modifications. Differential post-translational modifications on OPN confer distinct biological activity [32-34]. Because the Assay Designs capture antibody spans the site of major proteolytic cleavage, there is a potential that this assay can detect native C-terminal OPN fragment, which is not detected by the other assays. This is of interest given the recent demonstrations of C-terminal OPN fragment influencing cell migration and invasion [35], in some cases via an interaction with cyclophilin C [36]. Quantification of fl-OPN is also complicated by the potential for the protein to bind to cell surfaces via integrins, or to bind to circulating factors such as complement Factor H [37]. The consequences of protein-protein interactions on fl-OPN detection in ELISA assays are not known. Other tumor biomarkers, including prostate specific antigen, bind circulating proteins [38] that may affect detection ability. Secondly, since detection of early stage cancer is currently challenging, we hypothesized that if we could develop a more sensitive fl-OPN assay, it may be useful in detecting disease before advanced stages. Biologically, OPN expression has typically been shown to increase with tumor progression, suggesting it as a theoretical biomarker of cancer progression [6,7,39].

## Conclusion

We report the generation and characterization of novel anti-OPN MAbs, and their utility in an ELISA for fl-OPN. Compared to commercially available OPN standards and ELISA kits, we find that our novel ELISA has increased sensitivity to low levels of fl-OPN, and is accurate in quantifying known OPN standards. Similar to previous studies, our assay found elevated circulating fl-OPN levels in patients with metastatic breast cancer. The discrepancies between our results and previous quantification of plasma fl-OPN in breast cancer patients are significant, and difficult to resolve. We propose that several factors, including assay formats, OPN standards used, recognition of OPN isoforms, fragments, or differences in specificity, may contribute to the variations in fl-OPN amounts detected. Thus, further refinements of these assays may provide biological information regarding specific subsets of OPN species in cancer, whether generated by alternative splicing [40] or post-translational modification, including proteolysis.

## Abbreviations

OPN, osteopontin; ELISA, enzyme-linked immunosorbent assay; SIBLING, small integrin binding ligand N-linked glycoprotein; MMP, matrix metalloproteinase; MAb, monoclonal antibody; PBS, phosphate buffered saline; BSA, bovine serum albumin; fl-OPN, full-length OPN, PR, progesterone receptor; ER, estrogen receptor

## Competing interests

The author(s) declare that they have no competing interests.

## Authors' contributions

HSD and SS designed and performed the initial mouse immunization, monoclonal antibody generation, and screening for positive clones. HSD characterized select monoclonal antibodies and initiated ELISA. AP designed and optimized the final ELISA strategy, screened human samples, prepared and analyzed data, and drafted the manuscript. FLL led the design and statistical analysis of the human samples and contributed to manuscript preparation. SM led the study design, patient recruitment, and analysis phases of the cancer study, and contributed to manuscript preparation. A-KN provided daily supervision, design, and interpretation of the immunology work and assay development, and assisted in the preparation of the manuscript. LL, with A-KN, conceived of the study, was responsible for its overall design and implementation, and contributed to manuscript preparation. All authors have approved of the final manuscript.

## Pre-publication history

The pre-publication history for this paper can be accessed here:


